# Supplementary material for: Racial Difference in the Association of Long-Term Exposure to Fine Particulate Matter (PM2.5) and Cardiovascular Disease Mortality among Renal Transplant Recipients
Source: Int J Environ Res Public Health. 2021 Apr 18;18(8):4297. doi: 10.3390/ijerph18084297 (PMC8073484; doi:10.3390/ijerph18084297)
Supplement: Supplementary file 1 [file ijerph-18-04297-s001.zip › ijerph-1184134-SI.pdf]

Table S1: Multivariable adjusted hazard ratios for fatal events per 10 μg/m³ increment of $PM_{2.5}$ by Race with and without Adjusting for Socioeconomic

Outcome (Mortality)	Race			
	Black	%	Non-Black	
	HR (95%CI) *	Change	HR (95%CI) *	% Change
***All-cause Mortality*[¥]**				
Single Pollutant Full Model*	2.69 (2.28, 3.18)	Reference	2.27 (2.07, 2.48)	Reference
Single Pollutant Full Model + Proportion of Persons Below Poverty Estimate	2.61 (2.21, 3.09)	-2.97	2.24 (2.05, 2.46)	-1.32
Single Pollutant Full Model + Proportion of Civilian (Age 16+) Unemployed Estimate	2.74 (2.32, 3.24)	1.86	2.28 (2.08, 2.49)	0.44
Single Pollutant Full Model + Per Capita Income Estimate, 2006-2010 ACS	2.63 (2.23, 3.10)	-2.23	2.21 (2.01, 2.42)	-2.64
Single Pollutant Full Model + Proportion of persons with no high school diploma (age 25+) Estimate	2.71 (2.29, 3.20)	0.74	2.26 (2.06, 2.48)	-0.26
Single Pollutant Full Model + all 4 above SES Factors	2.78 (2.35, 3.29)	3.35	2.31 (2.10, 2.53)	1.76
***Total CVD Mortality*[¥]**				
Single Pollutant Full Model*	1.82 (1.33, 2.49)	Reference	1.87 (1.55, 2.25)	Reference
Single Pollutant Full Model + Proportion of Persons Below Poverty Estimate	1.76 (1.28, 2.42)	-3.30	1.85 (1.53, 2.22)	-1.07
Single Pollutant Full Model + Proportion of Civilian (Age 16+) Unemployed Estimate	1.88 (1.37, 2.58)	3.30	1.88 (1.56, 2.26)	0.53
Single Pollutant Full Model + Per Capita Income Estimate, 2006-2010 ACS	1.79 (1.31, 2.46)	-1.65	1.82 (1.51, 2.20)	-2.67
Single Pollutant Full Model + Proportion of persons with no high school diploma (age 25+) estimate	1.80 (1.31, 2.47)	-1.10	1.84(1.52, 2.22)	-1.60
Single Pollutant Full Model + all 4 above SES Factors	1.89 (1.38, 2.60)	3.85	1.87 (1.55, 2.26)	0.00
CHD Mortality				
Single Pollutant Full Model*	4.60 (2.10, 10.12)	Reference	1.88 (1.27, 2.79)	Reference
Single Pollutant Full Model + Proportion of Persons Below Poverty Estimate	4.61 (2.10, 10.12)	0.22	1.88 (1.27, 2.79)	0.00
Single Pollutant Full Model + Proportion of Civilian (Age 16+) Unemployed Estimate	4.97 (2.28, 10.83)	8.04	1.92 (1.30, 2.86)	2.13
Single Pollutant Full Model + Per Capita Income Estimate, 2006-2010 ACS	4.64 (2.12, 10.19)	0.87	1.89 (1.27, 2.79)	0.53
Single Pollutant Full Model + Proportion of persons with no high school diploma (age 25+) Estimate	4.80 (2.19, 10.51)	4.35	1.90 (1.28, 2.83)	1.06
Single Pollutant Full Model + all 4 above SES Factors	5.01 (2.29, 10.94)	8.91	1.99 (1.34, 2.96)	5.85

*Adjusted for all the following variables: sex, years after transplant, primary cause of ESRD, length in years from first ESRD services and first transplant, donor type, ESRD Network categories, BMI categories, and immunosuppressive medications. [¥] Effect estimates at attained age of 60 yrs. using time-dependent variables. [€] P-value for interaction term < 0.05.
